# The role of salivary biochemical markers and dental indices in the assessment of oral health of Egyptian children with cystic fibrosis: an exploratory study

**DOI:** 10.1038/s41598-025-25373-x

**Published:** 2025-11-18

**Authors:** Nehal Raid Salman, Engy Saad Elkaragy, Tamer A. Al-Shafie, Moustafa A. Matar

**Affiliations:** 1 Department of Pediatric Dentistry, Faculty of Dentistry, Pharos University in Alexandria, Alexandria, Egypt; 2 Department of Pediatrics, Faculty of Medicine, Alexandria University, Alexandria, Egypt; 3 Department of Oral Biology (Biochemistry), Faculty of Dentistry, Pharos University in Alexandria, Alexandria, Egypt

**Keywords:** Cystic fibrosis, Dental indices, Salivary biomarkers, Α-Amylase, SOD, Catalase, TNF-α, IL-6, Biochemistry, Diseases, Health care, Medical research, Microbiology

## Abstract

Although several studies have proven that cystic fibrosis (CF) does have a definite impact on oral and dental health, others have demonstrated that it does not. Here, we evaluated children with CF in the Egyptian population based on dental indices, salivary biochemical markers, and lifestyle factors, including diet/medications, in addition to routine bacteriological assays of plaque and caries specimens. Thirty-three children were classified into two groups: CF children (*n* = 18) and non-CF children (*n* = 15). Dental indices included DMFT and dmft indices to assess caries prevalence, plaque index (PI), and gingival index (GI) to evaluate periodontal health, along with developmental defects of enamel (DDE). Salivary biochemical markers, such as α-amylase enzyme, SOD, catalase, TNF-α, and IL-6, are used to assess oxidative/inflammatory status. Bacteriological analysis was conducted on oral samples. On comparing CF and non-CF children, salivary biochemical markers showed a significant increase in α-Amylase (*p* < 0.001), TNF-α (*p* < 0.001), and IL-6 (*p* < 0.001), and a significant decrease in SOD (*p* = 0.027) and catalase (*p* < 0.001). However, dental indices did not show any significant increase. Klebsiella spp. and Pseudomonas spp. were significantly higher (*p* = 0.026 and *p* < 0.001, respectively), whereas Streptococcus spp. and Moraxella catarrhalis levels did not differ. In conclusion, oxidative stress and inflammatory response in CF children extend to the oral cavity via saliva. However, routine medications appear to mitigate these effects, preventing periodontal alteration development.  Despite the elevated α-amylase activities, dental caries did not develop because of a low-carbohydrate diet.

## Introduction

 Cystic fibrosis (CF) is a rare, autosomal recessive disease. Several mutations occur in the CF transmembrane conductance regulator (CFTR) gene, located on chromosome 7, that are responsible for CF disease by affecting the CFTR protein function to regulate ions, particularly chloride and bicarbonate, and water transport across epithelial cell membranes, resulting in dehydration and increased mucus concentration of the lining cells. In particular, lung cells and pancreatic β-cells are the main affected epithelial cells^1–3^. This results in recurrent respiratory infections and diabetes, which are the two common conditions that accompany cystic fibrosis due to pulmonary afflictions and exocrine pancreatic insufficiency^4^.

Cystic fibrosis is associated with elevated levels of reactive oxygen species (ROS), leading to a state of oxidative stress. It is also characterized by chronic inflammation in the airways, driven by factors like bacterial infections and the release of inflammatory mediators from immune cells. Biomarkers like interleukin-6 (IL-6), tumor necrosis factor-alpha (TNF-α), and C-reactive protein (CRP) are often elevated in CF patients, indicating an inflammatory state^5^. Due to the high vasculature of the salivary glands, the exchange of such compounds from blood to saliva can occur either by passive or active transport, conveying oxidative stress and inflammation into the oral cavity and increasing the risk of developing various oral and dental alterations. The foregoing information draws attention to the oxidative stress and inflammation exerted by cystic fibrosis (CF) can be reflected in saliva, which underscores the utility of using saliva as a diagnostic tool for detecting both systemic and oral diseases^6^.

Various oral and dental alterations have been reported to accompany cystic fibrosis. Saliva has recently attracted attention as a non-invasive sample source that contains various biomarkers, which can aid in predicting oral health issues and assist in the diagnosis and monitoring of dental caries, gingivitis, and periodontitis^7^. The thickened secretions typical of CF can alter the composition and flow of saliva, leading to a dry oral environment that increases the risk of cavities, gingivitis, and oral infections^8^. Additionally, long-term antibiotic use, a common aspect of CF treatment, can result in changes to the oral microbiota^9^. Furthermore, CF patients may experience delayed dental eruption, enamel hypoplasia, and altered craniofacial growth patterns, requiring special considerations in dental care^10^. Recent studies have indicated that plaque scores in children with CF are comparable to those of healthy children and that CF children experience less gingival inflammation than control groups^11^. Previously, enamel hypoplasia and tooth discoloration were more prevalent in individuals with CF. Regular dental check-ups are essential for people with CF, as they are at an increased risk of developing these issues^12^.

Due to variability in data in the literature about CF, we conducted an exploratory study to establish an evaluation of the dental/oral health status of children with cystic fibrosis in our Egyptian population. In a distinguished manner, our evaluation encompassed the measurement of dental indices and the determination of salivary biochemical markers while taking into account the impact of diet and daily medications followed by such children.

## Patients, Materials, and methods

### Participants

**Fig. 1:**
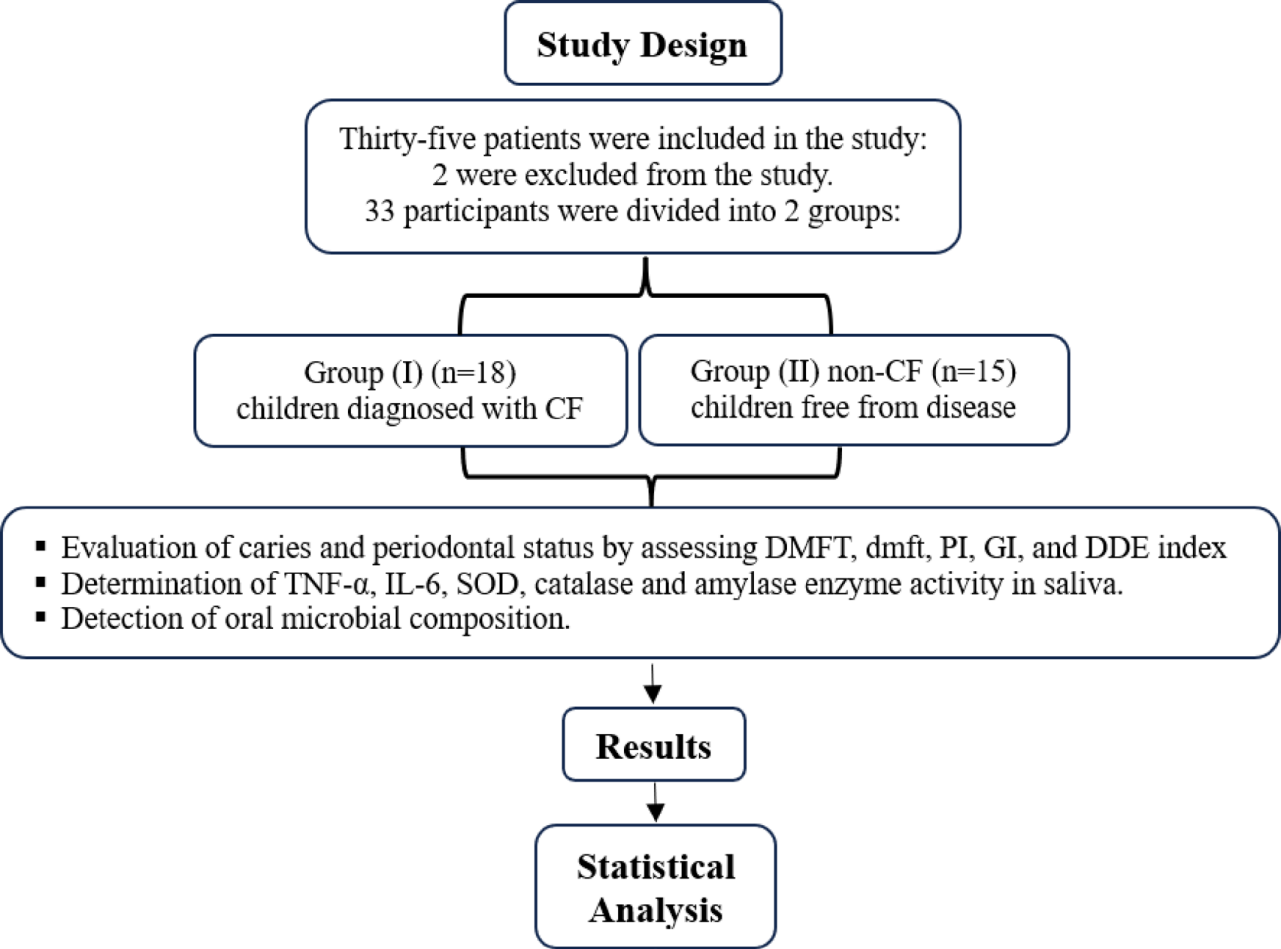
Study Design―Thirty-three children were enrolled in the study and divided into 2 groups: CF-group (*n* = 18) and non-CF-group (*n* = 15). All children were subjected to oral/dental examination to assess dental caries (DMF, dmft, and DDE indices), periodontal health (PI and GI indices). Children’s saliva was collected to determine antioxidant and anti-inflammatory, as well as amylase activity. Saliva cultures were performed to assess microbial composition.

Thirty-three children participated in this study, including 15 healthy (non-CF) children and 18 children with CF, all of whom were matched for age (*p* = 0.611), gender (*p* = 0.653), and socioeconomic status. The 18 children with CF were selected from the same pediatric clinic in Alexandria University Hospital where they were diagnosed. The 15 healthy (non-CF) children were chosen from the dental pediatric clinic and underwent a clinical examination before enrollment to rule out any systemic diseases. Figure 1 presents a flow diagram of the study design, children’s enrollment, and grouping.

### Inclusion and exclusion criteria

Children aged 5 to 11 years were included in both the control and patient groups. The control group (non-CF) consisted of healthy children, regardless of their history of dental issues, while the patient group included children with CF. Both groups followed the same exclusion criteria (e.g., recent antibiotics). Uncooperative children, those with systemic diseases, or had taken antibiotics within the six weeks before saliva collection were excluded.

### Sample size calculation

Sample size estimation was conducted with a 95% confidence level and 80% power. The caries experience was estimated to be 86.36% among patients with cystic fibrosis, compared to 27.27% among healthy controls. Based on a comparison of independent proportions, at least 12 participants were needed in each group. To account for potential non-response bias, this number was increased to 15 per group. Total sample size = Number per group × Number of groups = 15 × 2 = 30 patients. The sample size was based on Rosner’s method^13^, calculated by G*Power 3.1.9.7^14^.

### Oral examination and dental indices scoring

Thorough oral examinations were performed for the enrolled children in a dental chair using an examining light. Four dental indices were used in this study to assess caries prevalence and periodontal status in each child: the caries index for primary and permanent dentition, dmft and DMFT; respectively, MGI, PI, and DDE index. An assessment of intra-examiner agreement for the caries index using Kappa statistics was conducted on 15% of the sample. The kappa values for primary or permanent dentition were 0.773, 1.00, and 1.00 for decayed “D”, missed “M”, and filled “F”, respectively *p* < 0.0001). The scoring of each index followed the methodology described by Matar et al. (2025)^15^. Briefly, the caries indices, including dmft and DMFT, indicate the prevalence of caries in an individual. They are determined by counting the number of decayed, missing due to caries, and filled teeth, with scoring ranges of 0–20 and 0–28, respectively. Plaque (PI) scoring criteria include no plaque, thin film, moderate, or abundant, with a range of 0–3. The gingival (GI) indices are used to evaluate periodontal health, with criteria including normal gingiva, mild, moderate, or severe inflammation and bleeding, with a range of 0–4. The developmental defects of enamel index (DDE) are used to assess enamel defects, categorized based on type, extent, and location, using scoring criteria with a range of 0–4, including normal, demarcated, diffuse opacities, quantitative hypoplasia, and other.

### Saliva sample collection

Unstimulated whole mixed saliva was collected from each participant, whether healthy (non-CF) or with CF, at a specified time (9:00 to 11:00 am) to minimize variability in saliva composition between children. The participants were instructed not to eat, drink, or brush their teeth for at least 2 h before sample collection. To collect approximately 3–5 mL of saliva, each child was asked to spit into a sterile cup for about 10 min. Each saliva sample, collected in a sterile cup, was then transferred into two sterilized Falcon tubes (50 mL) and transported to the laboratory in an icebox. Once in the laboratory, the samples were aliquoted and stored at − 80 °C for future assays.

### Salivary biomarker determination

The Falcon tubes were centrifuged at 4000 g for 20 min to remove debris and used for determining biomarkers. The levels of TNF-α, IL-6, and total protein content were determined using enzyme-linked immunosorbent assay (ELISA) kits (Elabscience, USA) and calculated in pg/mg protein using a standard curve specific for each parameter. The TNF-α and IL-6 levels were then normalized based on the salivary total protein content in pg/mg protein. Spectrophotometer assays (Abcam, China) were used to measure salivary amylase, catalase, and superoxide dismutase (SOD) enzyme activity. The results were calculated in U/mg protein using the equation: ΔA/t. For reliability, all assays were done in duplicate or triplicate.

### Oral microbial composition detection

For each patient, a sterile cotton swab was used to sample a culture from the buccal site of the gingival sulcus of the teeth. In addition, soft caries and dental plaque samples were also collected. A parallel nasopharyngeal aspirate was taken from each patient at the same setting. All samples were sent concomitantly to the microbiology lab within one hour of sampling to be cultured on blood agar, chocolate agar, MacConkey’s agar, and Sabouraud dextrose agar.

### Statistical analysis

Data was analyzed using IBM SPSS for Windows, version 23, Armonk, NY, USA. Normality of quantitative variables was checked, including age and salivary biomarkers, using the Shapiro-Wilk test, and normal distribution was confirmed. All dental status indices (DMF, dmf, DDE, PI, and GI) were not normally distributed. Comparison between groups regarding age and all salivary biomarkers was done using an independent t-test, while the Mann-Whitney U test was employed for dental indices comparisons. The Pearson Chi-Square test was used to compare gender, dentition, diabetic condition, and DDE categories between groups. All tests were two-tailed, and the significance level was set at *p* < 0.05.

## Results

The CF and non-CF groups were matched in gender and age. Non-significant differences were obtained when comparing age and gender in both groups (*p* = 0.611, *p* = 0.653, respectively), Table 1. Of the thirty-three children who were recruited for this study, there were 11 (61.1%) females and 7 (38.9%) males in the CF children, while there were 8 (53.3%) females and 7 (46.7%) males in the non-CF children. The mean age of the CF children was 7.61 ± 2.23 years, while that of the non-CF children was 7.2 ± 2.37 years.

**Table 1:** Demographic data, dentition status, caries experience and diabetes history of the enrolled children with and without cystic fibrosis.

		CF children (*n* = 18)	Non-CF children (*n* = 15)	*p* value
Age in years	Mean ± SD	7.61 ± 2.23	7.20 ± 2.37	0.611
Gender: n (%)	Males	7 (38.9%)	7 (46.7%)	0.653
	Females	11 (61.1%)	8 (53.3%)	
Dentition: n (%)	Primary	5 (27.8%)	7 (46.7%)	0.261
	Mixed	13 (72.2%)	8 (53.3%)	
Caries experience: n (%)	Yes	11 (61.1%)	13 (86.7%)	0.101
	No	7 (38.9%)	2 (13.3%)	
Diabetes history: n (%)	Normal	11 (61.1%)	15 (100%)	0.025*
	Diabetic	4 (22.2%)	0 (0%)	
	Prediabetic	3 (16.7%)	0 (0%)	
	No	7 (38.9%)	2 (13.3%)	

### Dental and medical history and lifestyle

As shown in Table 1, the study showed that among children with cystic fibrosis (CF), most had mixed dentition, while primary dentition was more common in non-CF children; however, this difference was not statistically significant (*p* = 0.261). Caries experience was observed in 61.1% of CF children and 86.7% of non-CF children, with no significant difference between the groups (*p* = 0.101). Even non-diabetics, patients with cystic fibrosis, are often at risk of diabetes development; thus, it is meaningful to consider children with diabetes when dealing with such a medical condition^16^. Notably, diabetes-related conditions were present only in the CF group, which included four diabetic and three prediabetic children, leading to a significant difference compared to the non-CF group, where no such cases were recorded (*p* = 0.025).

Cystic fibrosis patients are vulnerable to frequently occurring complications; basically, diabetes in both adults and, to a lesser extent, children, and pulmonary disorders^16^. To manage diabetes or the risk of diabetes development, all our CF children were put on a high-fat/high-protein/low-sugar diet. To manage pulmonary complications, 3 to 5 chest physiotherapy therapeutic sessions were performed as a daily routine to improve breathing and reduce the risk of infection. Also, nebulization with hypertonic saline, salbutamol, and acetylcysteine was used to improve mucous clearance and lung function, as well as bronchodilators to relax airway muscles, in addition to antibiotics to manage lung infections Table 2.

**Table 2:** Diet, physical therapy, and medication history of children with cystic fibrosis.

Diet:	High-fat/protein diet Low sugar	
Physical therapy:	Chest physiotherapy	3–5/day
Medications:	Nebulization: hypertonic saline, salbutamol, (+/-) acetylcysteine	3–5/day
	Inhaled bronchodilators and corticosteroids	Intermittently
	Antibiotics (according to culture): Inhaled antibiotics Azithromycin	2/day 3days/week

### Dental and oral indices

**Fig. 2:**
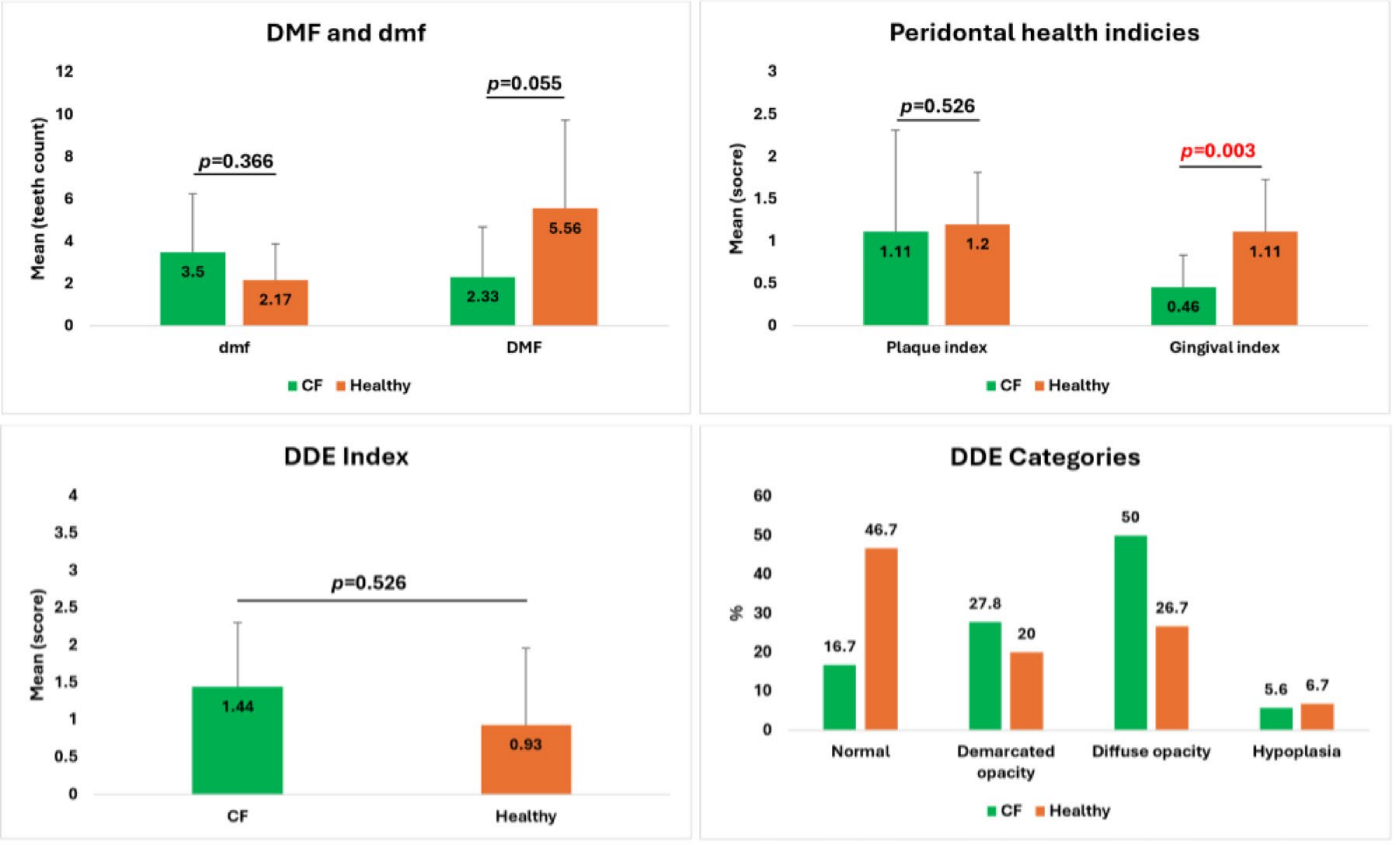
Dental Indices for the enrolled children, including caries index; DMFT and dmft (upper left), periodontal health indices; plaque index and gingival index (upper right) and developmental defects of enamel index (lower left) and its categories (lower right). Significant difference is considered at (*p* < 0.05) when the CF children group is compared with the non-CF children group.

The inter-examiner reliability was determined in the evaluation of caries status in either primary or permanent dentition. kappa-values were 0.87, 0.90, and 0.96 for decayed “D,” missed “M,” and filled “F,” respectively (*p* < 0.001). As illustrated in Fig. 2, dental and oral indices results revealed the following:

#### Caries index

Our results showed no statistically significant difference in the caries index between the CF and non-CF children groups. For primary dentition, the mean dmft index was 3.50 ± 2.74 in the CF group and 2.17 ± 1.72 in the non-CF group (*p* = 0.366). For mixed dentition, the mean DMFT index was 2.33 ± 2.35 in the CF group and 5.56 ± 4.16 in the non-CF group (*p* = 0.055).

#### Plaque and gingival indices

Similarly, our results revealed no statistically significant difference in the plaque index (PI) between CF children (1.11 ± 0.61) and non-CF children (1.20 ± 0.61) (*p* = 0.526). Interestingly, the gingival index (GI) was significantly higher in the non-CF group (1.11 ± 0.62) compared to the CF group (0.46 ± 0.38), with the difference being statistically significant (*p* = 0.003).

**Developmental Defects of Enamel (DDE) Index**: The results for the Developmental Defects of Enamel (DDE) index showed no statistically significant difference between CF children (mean: 1.44 ± 0.86) and non-CF children (mean: 0.93 ± 1.03) (*p* = 0.526). The distribution of DDE categories revealed that 46.7% of non-CF children and 16.7% of CF children had normal enamel. Demarcated opacity was observed in 20.0% of non-CF and 27.8% of CF children, while diffuse opacity was found in 26.7% of non-CF and 50.0% of CF children. Hypoplasia was present in 6.7% of non-CF and 5.6% of CF children.

### Salivary biochemical markers

**Fig. 3:**
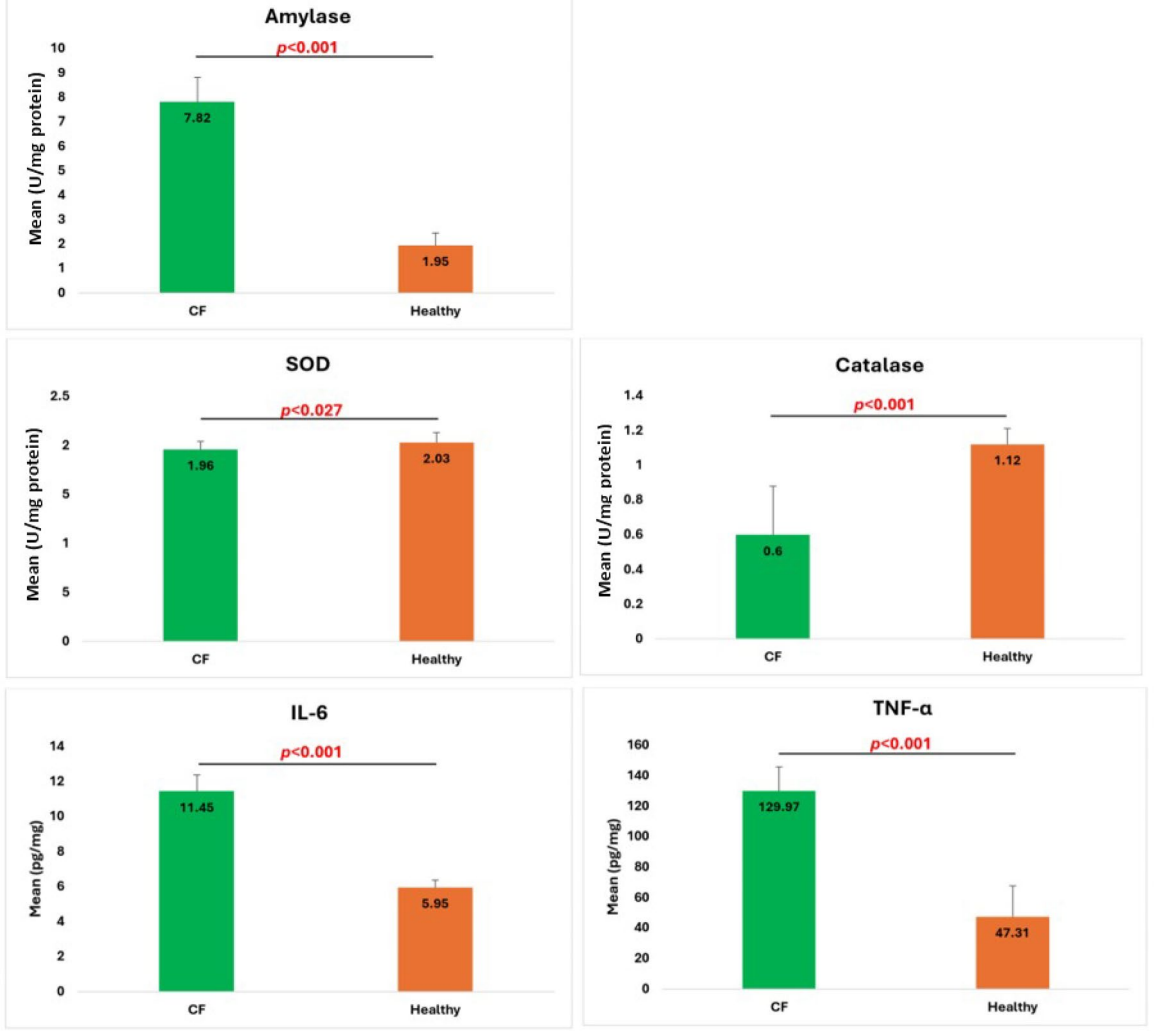
Salivary biochemical markers, including amylase enzyme activity (upper), antioxidant enzyme activities—superoxide dismutase (middle left) and catalase (middle right)—and inflammatory cytokine concentrations, such as interleukin-6 (lower left) and tumor necrosis factor-α (lower right). The results are presented as mean ± SD. A significant difference is considered at (*p* < 0.05) when CF children are compared with controls (non-CF children).

As illustrated in Fig. 3, the salivary biochemical markers results showed the following:

#### Amylase enzyme activity

Our results showed that salivary amylase activity was significantly higher in children with CF (7.82 ± 0.99) compared to non-CF children (1.95 ± 0.49), with the difference being statistically significant (*p* < 0.001).

#### Antioxidant enzyme activity

Our results revealed that the activity levels of superoxide dismutase (1.96 ± 0.08) and catalase (0.60 ± 0.28) were significantly lower in CF children compared to non-CF children (2.03 ± 0.10, *p* = 0.027, and 1.12 ± 0.09, *p* < 0.001, respectively).

#### Pro-inflammatory cytokine levels

Our results revealed that the levels of pro-inflammatory cytokines interleukin-6 (IL-6) and tumor necrosis factor-α (TNF-α) were significantly higher in CF children (11.45 ± 0.91 and 129.97 ± 16.05, respectively) compared to non-CF children (5.95 ± 0.44 and 47.31 ± 20.38, respectively), with both differences being statistically significant (*p* < 0.001).

### Oral microbial composition

Table 3 illustrates the number, type, and percentages of bacterial growth in oral cultures of CF children and non-CF children’s groups. A non-significant difference in the number of cultures showing Streptococcus spp. and Moraxella catarrhalis growth when comparing the two groups (*p* = 1.000 and *p* = 0.290, respectively), whereas the number of cultures showing Pseudomonas spp. and Klebsiella spp. growth was significantly higher in the CF children group compared to the non-CF children group (*p* < 0.001 and *p* = 0.026, respectively).

**Table 3:** Oral microbial composition of the enrolled children with and without cystic fibrosis.

	CF children	Non-CF children	*p*-value
Klebsiella spp. n (%)	9 (50.00%)	2 (13.33%)	0.026
Streptococcus spp. n (%)	18 (100.00%)	15 (100.00%)	1.000
Pseudomonas spp. n (%)	12 (83.33%)	1 (6.66%)	0.000
Moraxella catarrhalis n (%)	10 (55.60%)	11 (73.33%)	0.290

## Discussion

The impact of cystic fibrosis as a systemic disease on pediatric oral health has been studied in several previous reports^17–19^. However, the comprehensive view in the assessment of the oral and dental status for children with CF still seems modest. In the current study, we integrated more than one approach for accurate interpretation and evaluation of the oral/dental status in children with CF, including: dental indices measurement, salivary biomarkers determination, and consideration of diet/medication. Through measuring dental indices and determining salivary biochemical markers, our results suggest that daily diet and the routinely used medication regimen might inadvertently prevent children with CF from the onset of oral and dental alterations.

Dental indices are the most commonly used tools to quantify oral and dental diseases, allowing the interpretation of disease severity and treatment efficacy^20^. Regarding dental caries, although controversial information was detected in other studies in children with CF^9,17^, our results confirm, in agreement with the majority of researches that dental caries prevalence is fairly similar in both primary and mixed dentition, as illustrated by a non-significant difference in dmft and DMFT indices; (*p* = 0.366 and *p* = 0.055 respectively) when CF and non-CF children were compared. The periodontal health of children with CF was also discussed in other previously published articles, showing another controversy regarding this aspect^11,21^. In our study, we did not record a significant difference in PI index between the CF and non-CF children (*p* = 0.526), indicating that the amount and location of plaque are rather equal in both groups. On the other hand, we recorded an unexpectedly significantly lower score in the GI index in the CF children group in comparison to non-CF counterparts (*p* = 0.003), indicating a lower prevalence of gingival inflammation signs, including either color change, swelling, bleeding, or ulceration. The literature shows distinct variation in the enamel abnormalities among children with CF. In a review article, Pawlaczyk-Kamieńska T et al. 2019 reported three studies illustrating different results regarding tooth enamel abnormalities. Of the three studies, two showed a non-significant difference in DDE index between CF and non-CF children, whereas the third study recorded a significantly higher prevalence of enamel defects in CF children^21^. In total, our results didn’t record a statistically significant difference in DDE index categories, including normal enamel, demarcated opacity, diffuse opacity, and hypoplasia between the CF and non-CF children (*p* = 0.526).

Salivary biochemical markers play a crucial role in evaluating oral/dental as well as systemic conditions. They are frequently linked to caries, periodontal disease, and oral cancer, as well as diabetes and cardiovascular diseases^22^. In this study, we selected a panel of biochemical markers, including α-amylase, superoxide dismutase, catalase, IL-6, and TNF-α. The α-amylase is a digestive enzyme that represents a highly rich component of saliva. It contributes to the development of caries due to its ability to break down carbohydrates into disaccharides and short-chain oligosaccharides^23^. Our results showed a highly significant increase in α-amylase activity level in CF children in comparison to non-CF children. Although the impact of cystic fibrosis, as a disease, on α-amylase activity is actually beyond the scope of this article, it is worth noting that two old publications recorded an increased level of the enzyme activity in CF patients^24,25^, while decreased levels were reported by a recent study^26^. Since increased α-amylase activity is frequently correlated with increased occurrence of dental caries^27^, it was predicted that this significant increase would be accompanied by high caries index scores, but unexpectedly, this did not occur. This contradiction could be attributed to the low-carbohydrate diet, which permits only a small amount of carbohydrate to be exposed to the digestive enzyme action. Furthermore, frequent use of antibiotics for recurrent respiratory infections reduces the risk of dental caries development by inhibiting the growth of cariogenic bacteria.

Saliva contains several biochemical markers that can detect oxidative stress and inflammation at the molecular level, thereby enabling the prediction of periodontal disease during its early stages^28^. For this purpose, we used a panel of markers consisting of two antioxidant enzymes and two proinflammatory cytokines to be estimated in the saliva of both CF and non-CF children. Our results revealed that the activity levels of superoxide dismutase (SOD) and catalase in saliva, which act to prevent ROS accumulation and periodontium damage, were significantly lower in CF children in comparison to non-CF children, *p* = 0.027 and *p* < 0.001, respectively. Such decreased activities would be expected to stimulate a state of oxidative stress in the oral cavity, paving the way for the occurrence of different periodontal diseases. Furthermore, the oxidative damage to oral tissues is a pivotal event that ultimately leads to the development of chronic inflammatory disorders^28^. In agreement with the previous fact, our study recorded a highly significant increase in the pro-inflammatory cytokines, IL-6 and TNF-α levels in the saliva of CF children in comparison to non-CF children, *p* < 0.001. Strikingly, our study didn’t reveal any statistically significant signs of periodontal disease as indicated by PI and GI index scores. Considering the anti-inflammatory effect of the daily routine oral medication regimen, including nebulizers, bronchodilators, and corticosteroids, such medications most probably provided a continuous alleviative effect counterbalancing the inflammatory effects exerted by the systemic disease in CF children. However, long-term use of such medications has been reported to reduce adverse effects on adults’ oral health^29^.

Cystic fibrosis primarily affects the lungs and respiratory airways, resulting in thick, sticky mucus formation, providing a suitable environment for various types of bacterial growth^30^. Our results revealed that bacteria that impact CF children’s respiratory health, including *Klebsiella spp.*,* Streptococcus spp.*,* Pseudomonas spp.*,* and Moraxella catarrhalis*, are not only colonized in the mucous but also in dental plaques and caries. Therefore, dental plaques must also be considered a specific reservoir for bacterial colonization, as well, which undoubtedly worsens the condition. Our study strongly recommends the use of oral hygiene to scavenge bacteria colonized in plaque and caries, preventing recurrent respiratory infections. Additionally, it warns from the long-term use of nebulizers, bronchodilators, and corticosteroids to prevent periodontal alterations in the adult stage.

In conclusion, the current study illustrates that the effect of cystic fibrosis-linked systemic oxidative stress and inflammation is extended to the oral cavity, as indicated by the significantly decreased salivary antioxidant enzyme activity levels, including SOD and catalase, and the significantly increased levels of salivary proinflammatory cytokine levels, including TNF-α and IL-6, in our CF children. Although systemic oral oxidative stress and inflammation have been reported to be accompanied by oral/periodontal disorders, periodontal index scores of our CF children, including PI and GI, revealed a non-significant difference, suggesting a mitigating role of the daily routine medication regimen, which is known to exert antioxidant and anti-inflammatory effects. However, as reported, this beneficial effect can be reversed by long-term use of these medications. Furthermore, the significantly increased levels of salivary α-amylase activity were not accompanied by dental caries as indicated by the non-significant difference in dmft and DMFT caries index, drawing attention to the low-carbohydrate diet, which masks the effect of the high salivary α-amylase activity levels.

## Study limitation

Since cystic fibrosis is a rare disease, the major limitation encountered in this study was the relatively small sample size due to the small number of children with cystic fibrosis attending the pediatric clinic, Faculty of Medicine, Alexandria University. As a result, the interpretability and generalizability of the findings could be somewhat limited, and studies with larger sample sizes involving other Egyptian institutions’ pediatric clinics are essential to be conducted. Nevertheless, it is worth noting that all available pediatric patients attending the clinic were included in the current study. Besides, the determination of salivary flow rate is an important investigation; however, it was difficult to perform such an investigation, as CF children always have viscous saliva due to large amounts of sputum mixed with saliva as a result of the recurrent upper and/or lower respiratory tract infection accompanying CF-affected children. Other minor limitations include the uncooperative children who cannot spit saliva for lab investigation or are fearful of clinical examination for measuring various dental indices, in addition to children residing in rural and remote areas

## Data Availability

The authors confirm that data supporting the findings of this study, including high-resolution figures, are available upon request. Please contact the corresponding author, nehal.raid@pua.edu.eg.
